# Experiences of Older Spousal Caregivers for Caring a Person with a Memory Disorder

**DOI:** 10.3390/healthcare8020095

**Published:** 2020-04-13

**Authors:** Riitta Turjamaa, Johanna Salpakari, Liisa Koskinen

**Affiliations:** 1 School of Health Care, Savonia University of Applied Sciences, 70210 Kuopio, Finland; liisartkoskinen@icloud.com; 2 Clinical Nurse Specialist, Kuopio University Hospital, 70210 Kuopio, Finland; Johanna.salpakari@kuh.fi

**Keywords:** formal care, informal care, memory disorders, multiprofessional cooperation, peer support, spousal caregivers, qualitative research

## Abstract

Memory disorders have become a major public, social, and health concern among the aging population, and many of those who are affected are cared for at home by their spouse. The aim of this qualitative study was to describe the individual experiences of 10 older caregivers who were looking after a spouse with a memory disorder in 2016. Data were collected from volunteers who were recruited from a memory clinic at a Finnish health center using the thematic interview method and processed using inductive content analysis. The participants were six female and four male caregivers who had been married for over 10 years. The results indicated that caring for a spouse with a memory disorder involved a number of factors. These included the impact of social networks and changes in their everyday life, collaboration with service providers, and the caregivers’ well-being. However, our study showed that caregivers felt that the formal multiprofessional services they received were fragmented, which means that they were less likely to provide a holistic approach to caregivers’ situations. Better multiprofessional cooperation is needed in the community, including services such as memory clinics, home care and practical services, day centers, and short-term respite in care homes.

## 1. Introduction

Life expectancy is increasing in all developed countries, and the number of older people who retain their functional abilities, with no major health limitations, has increased [1,2]. Ensuring that older people can live independently in their own home for as long as possible is more topical than ever and a central target of national health policies across the globe. At the same time, the number of older people with progressive memory disorders is increasing, and this poses a real challenge for public health professionals and puts more pressure on health and social care funding and resources [3,4,5]. Figures from the Alzheimer’s Association (2019) show that 44 million people are suffering from Alzheimer’s disease or other types of progressive memory disorders worldwide and almost eight million patients are diagnosed each year. This number is expected to rise to more than 135 million by the year 2050. 

The Finnish Institute for Health and Welfare (2020) has reported that approximately 190,000 Finnish residents have some form of memory disorder and there are about 14,000 new cases of dementia each year. Progressive memory disorders can often lead to dementia, which is characterized by a decline in memory, language, problem-solving, and other cognitive skills. These affect a person’s ability to perform everyday activities [6,7]. Most people with a memory disorder have good functional ability and prefer living at home with their families [1,2]. However, progressive memory disorders have severe consequences for the everyday lives of both the person who has been diagnosed and their loved ones. The help and support they need with daily activities often increases dramatically as the memory disorder progresses [8].

Families play a significant role in caring for older people with memory disorders [8,9], in particular spouses [10], who provide the majority of this type of home care. This responsibility often continues into the advanced stages of the memory disorder and may include daily care, providing pharmacological and nonpharmacological treatment, and promoting general health, well-being, and quality of life [11,12]. 

Based on earlier studies, caring for a spouse with a memory disorder can have numerous effects on the caregiver’s overall well-being and the consequences can be diverse and complex [13,14]. As well as managing symptoms and behaviors related to their loved one’s memory disorder, caregivers have to make decisions about end-of-life care, including life-sustaining treatment [10,15]. Spousal caregivers have been reported to experience more depression and anxiety related to emotional, physical, and financial challenges than other family members involved in caring [16,17]. It has also been reported that they display greater cognitive and physical decline than other family members. This is because their role is time-consuming and includes tasks that may be very demanding from both a physical and mental point of view [18]. 

In addition, as their spouse’s memory disorder worsens it gradually increases the responsibilities and caregiving burden [11]. It has been reported that the health and social service sectors expect caregivers to take on responsibilities without assessing their ability to cope with them. Some caregivers have said they felt pressured to agree to performing those roles [19,20]. On the other hand, some people have reported that caring for their own spouse increased their satisfaction and well-being and gave greater meaning to their lives [8,11].

From an ethical point of view, it is very important to recognize that spousal caregivers have the same needs and autonomy as other service uses when decisions are made and goals are set with regard to their caregiving role [21,22]. These goals should be agreed in collaboration with community service providers and relatives. Research has shown that more goals should be tailored to the individual needs of caregivers [8,10]. Therefore, healthcare professionals working in the community play an important role in recognizing the needs of spousal caregivers and identifying the resources they need to support them in their daily caregiving role [23].

Most of the more recent studies have focused on investigating family caregivers’ health and quality of life [8,11,14]. However, we need to understand and develop our knowledge about what it means to act as a spousal caregiver for a person with diverse needs. This will help us to find ways to support them. The aim of this study was to describe the individual experiences of older spousal caregivers who cared for a spouse with a memory disorder.

## 2. Materials and Methods

### 2.1. Study Design

This was a qualitative, thematic interview study of older spousal caregivers carried out by the rural region in Eastern Finland. The participants were all attending a primary care memory clinic on a voluntary basis, and one of the authors (J.S.) gave them a presentation about the study and asked if anyone would like to participate. Memory clinics have been set up across Finland following recommendations from an expert panel convened by the Finnish Alzheimer’s Disease Society. They provide peer support and are staffed by health and social care professionals who specialize in the diagnosis and treatment of memory diseases.

Those that volunteered to take part provided written, informed consent and were then contacted by phone to organize an interview. We chose to use thematic interviews as they enabled us to explore the personal and sensitive life experiences of spousal caregivers and find out what it was really like to look after a spouse with a memory disorder [24]. The caregivers who took part in this study were all looking after spouses who had advanced-stage memory disorders, such as Alzheimer’s disease and those associated with cerebrovascular disease. We have used the term memory disorder to cover all these conditions.

### 2.2. Participants and Data Collection

We recruited 10 older spousal caregivers—six females and four males—who spoke Finnish and were happy to volunteer to take part. They were 69–86 years old and their average age was 76.8 years. Six of them had acted as a spousal caregiver for one to two years, and four had been a caregiver for less than a year. The couples had all been married for at least 10 years at the time of the study (Table 2).

**Figure 1:**
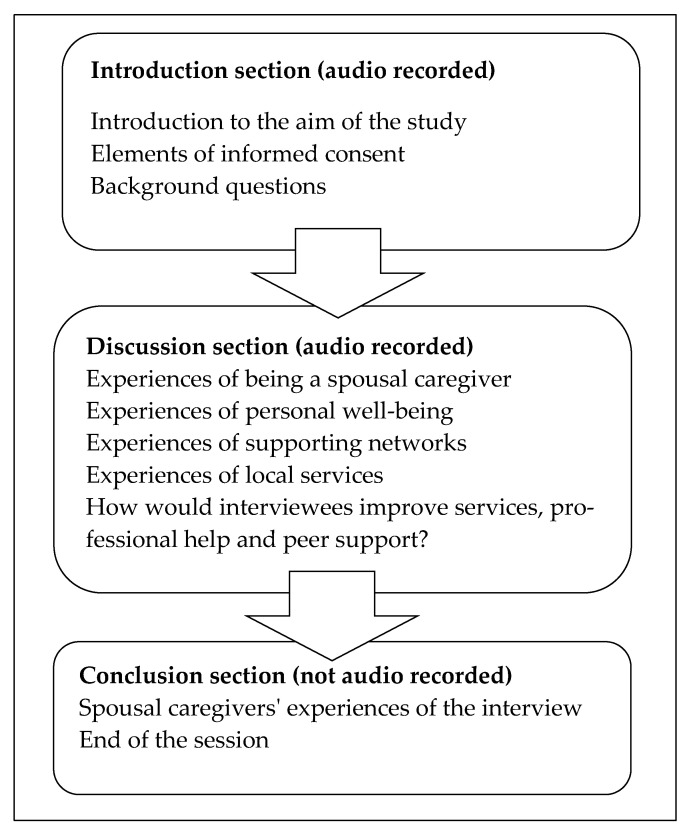
Thematic interview guide.

The individual interviews took place in a memory clinic, based in a health center in a town in Eastern Finland during spring 2016. They were conducted and recorded by the same researcher (J.S.). An interview guide was designed, consisting of an introduction, discussion, and conclusion, to help the interviewer take the participants through the process (Figure 1). The text in the introduction was used at the start of the interview, to remind participants of the voluntary nature of the study and that any comments they made would be reported in a way that respected their identity and need for personal confidentiality. The interviewer asked them about their background, such as their age, their spouse’s medical condition, and the support that they provided for them. Five themes were explored during the discussion section. The recorded interviews lasted for 25 to 50 min and after they had ended the participants were encouraged to talk about how they felt about the actual interview (Figure 1). Each interview was adjusted to the pace set by the interviewee.

### 2.3. Data Analysis

The main goal of the inductive content analysis technique [25,26] was to identify and describe the individual experiences of older spousal caregivers. The recorded data were transcribed by one researcher (J.S.). The first step was to modify the transcribed text into simplified sentences. Then, the data were placed in subcategories (e.g., the caregiver’s positive life attitude), upper categories (e.g., the empowering impact of social relationships that included the spouse with the memory disorder), and the main categories (e.g., spousal caregivers’ social network) (Table 1). The final phase of the analysis was conducted with the entire research group, as recommended by previous studies [25,26].

### 2.4. Ethical Considerations

The Research Ethics Committee needs to assess research plans when vulnerable individuals are involved in a study [27,28]. However, as this study focused on older spousal caregivers living independently in their own homes, no evaluation was required. Instead, appropriate permission was granted by the health center that ran the memory clinic that the participants and their spouses attended. All the spousal caregivers received verbal and written information about the study and were told that participation was voluntary and they could withdraw from the study at any time. Participants were told that the interview material was confidential and only the researchers had access to it. They were also told that the study results would be reported in such a way that their identify and confidentiality would be respected. After they were provided with information about the study, the participants were given time to consider whether they wanted to take part. Finally, written informed consent was obtained from all participants. Any data were kept in a password-protected computer.

## 3. Results

Demographic details of the spousal caregivers and their spouses are shown in Table 2. The couples were Finnish by ethnicity and lived all in the same rural town. Half of them lived in a detached house, and half in an apartment house. The spouses’ average age was 75 years and they had been teachers, private entrepreneurs, bank employees, shop assistants, and cleaners by profession.

**Figure 2:**
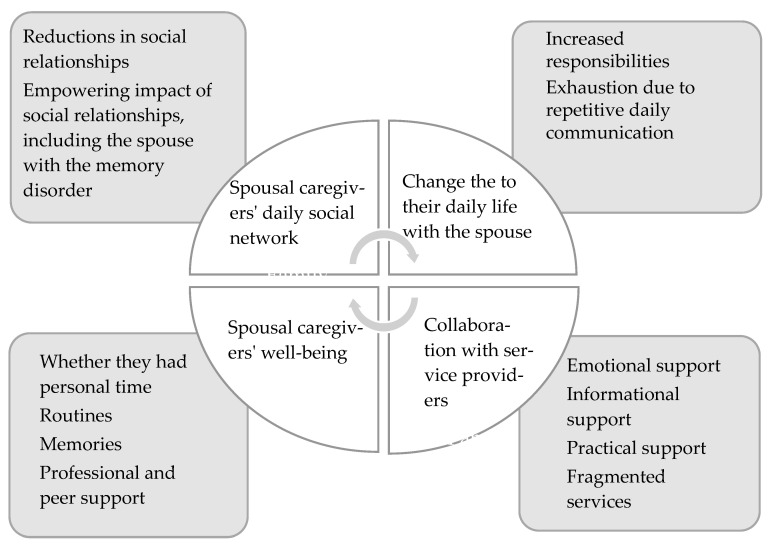
Upper and main categories describing spousal caregivers’ experiences.

Based on our results, the individual experiences reported by older people caring for a spouse with a memory disorder consisted of their daily social networks and the changes to their daily life with their spouse. They also included collaboration with service providers and their own well-being (Figure 2). We illustrate the results with the original quotes of the interviewees.

### 3.1. Daily Social Networks

The caregivers’ social network could be seen in two ways: the challenging reduction in social relationships with other people or the empowering impact of remaining relationships, including the spouse with the memory disorder. They said that their social network was essential for their quality of life, but they found that these became smaller because old friends started to deliberately avoid the couple. Some people found it too hard for them to have contact with an old friend who became dramatically different, due to their memory disorder. In addition, the person with the memory disorder could behave in a very odd way and this tended to push friends away. People with memory disorders had little interest in outside events. They felt safe in their own home, where they had control over their life. In contrast, the outside world was confusing and frightening.

*“Neighbors started to avoid us. Gradually, my husband became feared of all external action. He also lost his willingness to visit friends. Home is good, he says. …. Our social network has shrunk.”*

On the other hand, the spousal caregivers said that they were surrounded by an empowering social network. They also explained how important their own positive life attitude was to relationships. Meeting friends or taking part in events outside home provided them with a stimulating alternative to a daily life that was filled with limitations. They felt that if they were open about their spouse’s memory disorder it was liberating, as it improved the quality of their social network. The interviewees said that the history they shared as a married couple was an empowering element and present in everything they did together. They could not imagine a future without their spouse and felt that they had an obligation to care for them now they had a memory disorder. They also said that carrying out daily routines together empowered them both and helped their spouse to retain their identify and feel they were making an important contribution. The caregivers were empowered by how thankful their spouse was for the care they received and a small gesture or a beautiful word had tremendous power and gave them renewed strength to carry on.

*“He always reminds me of his satisfaction. He says, I am lucky you are here. …He hugs me and says you tolerate me as I am. … Well, that is how it goes. It is these thing that help me continue.”*

### 3.2. Changes in Daily Life with the Spouse

Despite the big changes that their spouse’s memory disorder had on their daily life, the caregivers said they were able to cope with just occasional external help. They had developed various techniques to increase their spouse’s safety when they had to go out. For instance, they used security cookers, door alarms, or written or illustrated information sheets to communicate important matters. However, their life was influenced by having greater responsibilities and they felt that daily communication could be exhausting, for example, because they frequently got asked the same questions.

*“This is my duty. When I look at him I see a helpless child. …. To this point our relationship has come. I am unhappy if he is sad and happy if he is glad. …But behind all is intimacy.… Often I hope he was healthy enough to ask how am I.”*

Greater responsibility meant having to be alert around the clock. The caregivers supervised, counselled, encouraged, controlled, and monitored how their spouse behaved to avoid them having accidents. They were also responsible for all the couple’s practical and financial matters. Their spouse became very dependent on them and became distressed as soon as they were not in their field of vision. Taking care of pressing matters outside the home often made caregivers very anxious. As time went on, some of their spouses became annoyed, discontent, and emotionless, but this was not always the case. Some became warmer and more thankful and this brought the couple closer together. As the memory disorder progressed, the couple’s relationship moved into a new phase, as their marital and sexual relationship was replaced by caregiving and closeness. Being able to pursue interests outside the home became dependent on whether external help was available. The interviewees saw this situation as an obligation and looked back on how their marriage used to be with longing. 

The memory disorder caused exhaustion in daily communication, as the sick spouse did not identify with either their symptoms or the consequences of the disease. They refused to receive any form of external help, even though this was an essential part of them staying at home with their caregiver. In contrast, the caregivers accepted this reality. As their love one’s short-term memory became worse it led to tiring situations and quarrels. The caregivers wanted to respect the spouse by responding to questions and explaining things repeatedly, but the spouse could become offended and angry.

*“Questions and questions. Repetition is emotionally exhausting and she is very sensitive. If I answer her questions a little impatiently, she says don’t yell at me, you are angry. I immediately realize she is offended.“*

### 3.3. Collaboration with Service Providers

The spousal caregivers collaborated with various professional service providers and peer support organizations and these provided emotional, informational, and practical support. The caregivers said that healthcare professionals did not pay enough attention to their emotional well-being as their spouse’s condition deteriorated. In addition, the services that were available did not meet the needs of caregivers. Services were fragmented by nature and provided by many different service providers. Caregiver fatigue was not properly recognized, which delayed them receiving suitable help and wore down their emotional resources.

*“Well, a lot of information has been given. … if a person knows nothing and is just getting started, it is impossible to understand the whole process. I need hand-to-hand guidance on the future of the patient and caregiver. I have to understand the causes and changes that will take place. … The caregiver must understand what the future will be. But this is confusing.”*

Having a spouse with a memory disorder caused various changes and problems in the caregivers’ everyday lives, as they needed more information from external organizations. These included care pathways, taking care of medication, day centers, respite care in care homes, and applications, such as video-based communication systems, that could be provided in their home. Peer support and professional guidance were both valued. However, caregivers became anxious when the amount of information was impossible to digest or professionals did not provide instructions in a timely manner.

Practical support consisted of being aware of services, like cleaning, transport, day centers, and respite care, as well as help to fill in an application for care allowance. These were essential in relieving the burden of daily life with the spouse at home. Lack of transport complicated daily life when the spouse lost their driving license and the caregiver did not drive. Being able to get their spouse a place in a day center meant the caregiver could have time to themselves or time to get other things done. As the memory disorder progressed rapidly, short-term respite care in a care home was valuable for the well-being of the interviewees. However, it was evident that the caregivers were hesitant to send their loved ones away from home.

*“We agreed (with the health center) that I would take my lady to a nursing home for a few days. But then I realized how lonely I would be myself. I think, I would suffer if I did.”*

### 3.4. Spousal Caregivers’ Well-Being

All the spousal caregivers said that their new role had changed their life. Because their job was to care for their spouse round the clock, exhaustion, loneliness, shortage of personal time, and lack of sleep had an impact on their well-being. Days were different and depended on what mood their spouse was in. Adequate support or help was not available in a timely manner. However, all of the caregivers had found ways to promote and maintain their own well-being. This consisted of making sure they had personal time, routines, memories, and professional or peer support.

Personal time improved the caregivers’ ability to cope with daily life and included activities and events outside the home or finding private time at home. These included women’s clubs and fitness groups or short visits to a summer cottage for private time. Being interested in the outside world, news, or art improved their well-being. Sometimes their well-being was connected to very small events, such as watching a movie or drinking a cup of coffee in peace.

*“There are days you get a minute to drink a cup of coffee in peace and you start to feel better. … In the evening, you will be amazed: Oh dear, this day is over and we survived. “*

Despite the obstacles they faced, the caregivers wanted to take care of their spouse at home for as long as their memory disorder allowed. Daily routines improved their well-being and these could include phone calls from friends and children who lived some distance away, a tailored day and reflecting on caregiver role. In addition, remembering how they lived as a couple before their spouse developed a memory disorder was important for their well-being and this included positive and negative aspects. They respected their common history, as it was the main reason why they identified themselves as family caregivers, not nurses.

*“This family caregiving is completely different thing than nursing. You are completely tied to what you do. … We have common history. We have been together for more than 50 years. This fact is constantly present, accept it or not. Well, if I speak frankly, sometimes I have a dream that someone in turn would take me in his arms.”*

Professional and peer support were acknowledged by the caregivers as important elements that improved their well-being. They mainly met professional staff when they were visiting community health centers or memory clinics alone or with their spouse. However, healthcare professionals did not always ask the caregivers about their well-being and that is why they preferred peer support.

*“Maybe they (healthcare providers) don’t think it is important to ask how I cope. … The person I would like to talk to would have done this job and would know what it is like.”*

## 4. Discussion

This study explored the individual experiences of older caregivers looking after a spouse with a memory disorder. It focused on daily social networks and changes in daily life with their spouse, collaborations with service providers, and the caregivers’ well-being.

The results indicated that the caregiver role had started slowly, a long time before their spouse was diagnosed and they became their official family caregiver. They regarded their caregiver status as a natural continuation of a long marriage. These spousal caregivers echoed research that reported that they played an immensely important role, from a humane perspective and economic point of view, as they supplement the provision of formal community care [29].

This discussion focuses on the two key observations that emerged from this research. The first observation was the collaboration between the spousal caregivers and the service providers. In our study, the spousal caregivers’ value and their care burden were not adequately considered by the formal or informal service systems that surrounded them. In addition, the professional and peer support that they received was not organized in an ideal way. Therefore, appeals from caregivers for recognition and visibility were sometimes ignored by the formal organizational structures that were meant to assist, help, and support them.

The spousal caregivers said that the formal help they needed was provided by fragmented, multiprofessional services and that they were coordinated by people they did not have direct contact with. They said their relationships with healthcare professionals were confidential and good, but this was not reflected in the services they were offered. The negative impacts of fragmented service provision on the quality of life and well-being of families caring for someone with a memory disorder has also been reported by previous studies [19,20]. Fragmented services can actually provide confusion rather than support if they do not take into account the specific help that caregivers need to be able to support their spouse on a daily basis.

Multiprofessional collaboration between service providers has been recognized as the best way to provide organized and seamless services. These have been characterized by shared information, collective decision-making, and appropriate support for family caregivers [19]. In addition, it is important that caregivers and their spouses play an active role in the multiprofessional planning groups that provide the services they need [14,18]. Caregivers are the best people to say what they need for themselves and the person they care for. Each of them can provide personal expertise that contributes to the service collaboration. If caregivers feel empowered, this may improve the care they can provide at home and delay the need for their spouse to be institutionalized.

Therefore, it is important to make sure that spousal caregivers are recognized as part of the task force that provides the person with a memory disorder with support from a well-organized, adequate, seamless, and individualized support model. Healthcare professionals who work with caregivers need specific competencies, such as the ability to work in partnership with caregivers and respect how the caregiver and their spouse want to live their lives. They should ensure that their encounters with caregivers feel unhurried. According to Garvey et al. (2019), family caregivers were empowered by easy-going counselling sessions with professionals, where they could discuss the memory disorder and its consequences and what community care support services were available.

The second observation was the changes in the daily lives of the caregiver and spouse. According to our results, caring for someone with a memory disorder round the clock caused severe problems. There were communication challenges between the couple, and the marriage had turned into a dependency–responsibility relationship. In addition, social networks had got smaller, and the caregivers suffered from emotional and physical exhaustion, such as anxiety, loneliness, and lack of sleep.

Previous studies by Aguilera et al. (2019) and Grano et al. (2017) showed that caregivers were only able to take a break from their responsibilities when they knew that their spouse felt well. This highlighted the need for flexible care models that alternate home care and periods of respite in a care home. However, the caregivers in our study said that the spouses did not want to participate in any day care activities or go into short-term respite care. Furthermore, the caregivers saw their role as an empowering obligation and did not want to persuade their spouse to change their mind if they were reluctant. When healthcare professionals encounter such situations they need the emotional sensitivity and negotiation skills to agree comprehensive solutions with the couple.

Our results support previous studies that stated that it was important to recognize high-risk carers [9]. We found that the risk was evident and caregiver exhaustion was often triggered by a sleepless spouse. This affected the caregivers’ ability to sleep and caused an emotional burden [13,30]. Being alert day and night, and deprived of proper sleep, has been shown to rapidly decrease the well-being of caregivers [11,12]. The quantity and quality of sleep is a major concern when a caregiver is looking after a spouse with a memory disorder and it is important that healthcare professionals discuss this at every meeting.

## 5. Study Limitations

This study has some limitations that warrant attention. The study was conducted among a small group of Finnish spousal caregivers of persons with a memory disorder, and the findings do not represent the view of all spousal caregivers in Finland. Therefore, the results cannot be generalized without further studies that are focused on identification of the role and well-being of older spousal caregivers. The qualitative nature of the study should be considered when assessing the trustworthiness of the results. However, participants enjoyed telling their experiences in confidence, and the large number of opinions among the number of participants gives confidence in the results. In pondering the benefits of this study, it was agreed that the results could possibly trigger discussion among health providers on the vulnerability of spousal caregivers.

## 6. Conclusions

The results of this qualitative, thematic interview study contribute to our understanding of the views and the challenges faced by older caregivers living in a Finnish rural town and caring for a spouse with a memory disorder. It highlights a number of elements that might inform how care providers and peer groups help couples in a similar situation. Service structures and provision vary between, and even within, countries. However, it is clear that most, if not all, community care organizations cannot respond to all of the challenges faced by caregivers looking after spouses with memory disorders. There is a need for overlapping, seamless, multiprofessional, and client centered support structures for such couples, such as formal and informal services. These could include memory clinics, home care, practical home support services, day care centers that provide activities, and short-term respite care in care homes. Peer support networks and voluntary organizations should also be incorporated into care and support networks in a more proactive way. Identifying older spousal caregivers who risk exhaustion is also essential, as well as alleviating the causes, such as sleep problems.

## Figures and Tables

**Table 1:** An example of data analysis.

Main Categories	Upper Categories	Sub-Categories	Examples of Simplified Sentences by the Spousal Caregivers
Spousal caregivers’ daily social network	Reductions in social relationships	Friends and neighbors avoid the couple Changes in the behavior of a person with memory disorders	“My husband’s behavior has chaged during the memory disorder. I think that our neighbors are afraid of my husband. It is so sad.”
	The empowering impact of social relationships	The caregiver’s positive life attitude The loved spouse with the memory disorder	“I enjoy meeting my friends. We’ll go to the cafe and have a chat, or they’ll come over to our home. Things that cheer up the mind.”

**Table 2:** Demographic details of the spousal caregivers and their spouses.

**Demographics of caregivers**			n	
*Gender*				
Female			6	
Male			4	
*Type of housing*				
Department house			5	50
Apartment house			5	50
	Mean		Min	Max
*Age*	76.8		69	86
	< 1 year		One year	Two years
*Length of caregiver status*	4		3	3
**Demographics of spouses with memory disorder**			n	
*Gender*				
Female			4	
Male			6	
	Mean		Min	Max
*Age*	75		66	84
*Length of memory disorder*	< 3 years 4		> 3 years 3	Over five years 3
	2 years	3 years	4 years	6 years
*Length of memory disorder*	1	3	3	3
